# HEE-GER: a systematic review of German economic evaluations of health care published 1990–2004

**DOI:** 10.1186/1472-6963-7-7

**Published:** 2007-01-12

**Authors:** David LB Schwappach, Till A Boluarte

**Affiliations:** 1 Research Institute for Public Health and Addiction, Konradrstrasse 32, 8031 Zurich, Switzerland; 2 Health Policy, Faculty of Medicine, University Witten-Herdecke, Alfred-Herrhausen-Str. 50, 58448 Witten, Germany

## Abstract

**Background:**

Studies published in non-English languages are systematically missing in systematic reviews of growth and quality of economic evaluations of health care. The aims of this study were: to characterize German evaluations, published in English or German-language, in terms of various key parameters; to investigate methods to derive quality-of-life weights in cost-utility studies; and to examine changes in study characteristics over the years.

**Methods:**

We conducted a country-specific systematic review of the German and English-language literature of German economic evaluations (assessment of or application to the German health care system) published 1990–2004. Generic and specialized health economic databases were searched. Two independent reviewers verified fulfillment of inclusion criteria and extracted study characteristics.

**Results:**

The fulltexts of 730 articles were reviewed of which 283 fulfilled all entry criteria. 32% of included studies were published in German-language. 51% of studies evaluated pharmaceuticals and 63% were cost-effectiveness analyses. Economic appraisals concentrate on few disease categories and important health areas are strongly underrepresented. Declaration of sponsorship was associated with article language (49% English articles vs. 29% German articles, p < 0.001). The methodology used to obtain quality-of-life weights in published cost-utility studies was very diverse, poorly reported and most studies did not use German patients' or community health state valuations.

**Conclusion:**

Many of the German-language evaluations included in our study are likely to be missing in international reviews and may be systematically different from English-language reviews from Germany. Lack of transparency and adherence to recommended reporting practices constitute a serious problem in German economic evaluations.

## Background

Economic evaluation of health care has evolved as an important tool for assessing the costs and benefits of health care and a large amount of evaluation studies has been published during the last decades. Several systematic reviews have been conducted that assessed the characteristics and quality of published studies [1-4]. The main conclusions that can be drawn from these studies is that adherence to methodological standards is increasing but still far from perfect. For example, in an analysis of pharmacoeconomic submissions to the Australian Pharmaceutical Benefits Scheme, 67% of submissions had significant problems [5]. The majority of problems identified (64%) were deemed avoidable. Neuman et al. report in an analysis of cost-utility studies that in only 73% of the studies published 1998–2001, the authors clearly presented the study perspective [1]. In a recent analysis of cost data in economic evaluations conducted alongside randomized controlled trials, only 37% of the reviewed evaluations presented a cost-effectiveness ratio or estimated net benefits and only 57% of these reported the uncertainty of this statistic [6]. However, these reviews have covered the English-language literature only, and analyses published in languages other than English are therefore systematically missing. However, as evaluation studies are highly context dependent, it is likely that many studies are being published in the languages of the countries whose health care systems have been addressed. These country-specific analyses, often published in national journals, may also have considerable impact on local decision makers. Until now, no systematic review has been conducted to assess growth, characteristics and quality of German economic evaluations. The aim of this study was to overcome this gap and to 1) analyze in which journals and in which language German economic evaluations have been published; 2) describe the characteristics of the studies in terms of various key parameters, such as type of evaluation, study design, covered diseases, study perspective, funding sources and others; 3) examine changes in study and publication characteristics over the years and 4) investigate methods to derive quality-of-life weights in cost-utility. While our study included all types of full economic evaluations, we paid special attention to cost-utility-analyses, the methods used to derive utilities for calculation of quality-adjusted life years (QALYs) in published studies and, in particular, whether preferences have been elicited from German patients or community members.

## Methods

### Literature search and study selection

We searched the databases Embase, Pubmed, Econlit (silverplatter), Cinahl, NHS-Pharmline, NHS EED, and OHE HEED for relevant articles. The searches were conducted in April 2005. In addition, the references of retrieved articles were manually searched for further material. The relevant volumes of a German-language health economic journal were completely hand searched for relevant articles as this journal is not systematically covered in international databases [7]. We also contacted a list of German health economists and recognized authors (n = 63) via email and asked to provide us their bibliographic data. Of those, 17 (27%) responded and sent their publication record and relevant material. As the use or translation of technical terms for indexing non-English literature in international databases is often inconsistent or errant we defined a search strategy with high sensitivity but low specificity [8,9]. The search strategy consisted of freetext and MeSH terms related to economic evaluation. The resulting hits were filtered for the occurrence of the terms "German" or "Germany" in any field. The retrieved records were further refined for the relevant year range and, where available, limited to journal articles. The search strings are provided in the Appendix (see Additional file 1). Studies were included in the review when they fulfilled all of the following inclusion criteria:

• Full economic evaluation, i.e., comparative analysis of costs and outcomes of at least two alternatives;

• Applied study (trial generating primary data or modeling of secondary data). Reviews, letters, abstracts, methodological and general articles were excluded;

• Assessment of or application to the German health care system;

• Journal articles, i.e., exclusion of books, HTA reports, grey literature;

• Published between 1990–2004 (i.e., after German reunification);

• Published in English or German-language

We did limit our analysis to evaluations published in official journals to assess those publications that have at least undergone some basic quality control. While, for example, some newer Health Technology Assessment reports may report original data analysis and would have therefore fitted our inclusion criteria in principle, they are usually not subject to external standardized peer review. It is also often hard to define what constitutes a HTA report since they may be published as grey literature by non-official agencies.

### Data extraction and critical appraisal

We developed a checklist comparable to that used by other researchers to extract data from the fulltexts alongside review [1,10]. We collected details on study objective, intervention type and disease category, design, methodology and background data such as sponsorship. The data extraction form did not include explicit quality ratings. As one of our interests was the investigation of health effects assessment in cost-utility studies, data relating to health and preference measurement methodology were extracted in detail. We recorded the health measurement instrument used, the technique used for valuing health states, and the groups of individuals from whom quality of life data and valuations were obtained. We also compared the extracted data relating to utility measurement and reporting of QALY calculation against criteria recommended by Stalmeier et al. [11] and Richardson and Manca [10]. These criteria include, e.g., reporting of elicitation procedure, sample size, anchor states, and others. For all studies, we used only the information provided in the original publication. However, in case the published methodology regarding health status measurement provided in cost-utility studies was not sufficiently detailed to extract the data as needed but authors referred to other studies as data source, these references were obtained to extract the relevant information. For example, if authors cited a second study as source for utilities, we obtained this article to determine whether this study contained information on utility elicitation. Each paper was independently read by a single researcher. A random sample of 10% of studies was assessed by two reviewers to determine interrater reliability for each item. Cost-utility studies were all reviewed by both reviewers. Reviewers were also allowed to defer studies in case they were uncertain about one or more items. These studies were assessed by a second reviewer and discussed in consensus meetings to resolve discrepancies. After critical appraisal, information collected in data extraction forms were transferred to an electronic database.

### Analysis of data

Kappa statistics (K) were calculated to assess interrater reliability for each item. We analyzed the distribution of extracted categories of economic evaluations and investigated differences between journal specialty (medical vs. health care sciences journals, as defined by the ISI Journal Citation Reports subject category listing) and German and English-language articles. To assess developments over time, we defined the year ranges 1990–1998 and 1999–2004. Comparisons were made using chi-square tests. A p-value of less than 0.05 was considered significant. All analyses were performed using STATA 9 software [12].

## Results

**Figure 1:**
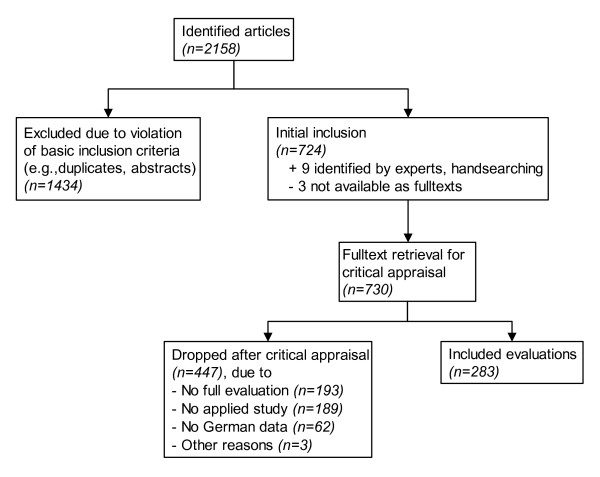
Overview of in- and exclusion of studies.

The systematic literature search initially identified 2,158 candidate articles, of which 724 were selected for fulltext retrieval (figure 1). The majority of articles were discarded at this initial stage mainly because they were duplicates, or it was obvious from the bibliographic data that they violated basic inclusion criteria. Further 9 articles were identified through hand searching or submitted by the contacted experts. 3 articles were not available as fulltexts, neither from the publisher or document suppliers nor directly from the authors. In summary, the fulltexts of 730 articles were reviewed. 447 articles were dropped after the critical appraisal because they failed one or more inclusion criteria. Interrater reliability (K) was assessed on a sample of 90 studies (10% random sample + all remaining 17 CUAs not included in this sample by random). K was 0.978 (95% CI 0.934 – 1.0) for study inclusion, i.e., fulfillment of all entry criteria. K for all other items ranged from 0.921 (95% CI 0.885–1.0) for primary disease category to 1.0 for the items 'study design' and 'study setting'. Overall, a very good level of agreement was observed [13]. 283 studies fulfilled all entry criteria.

### Basic characteristics of included studies

**Figure 2:**
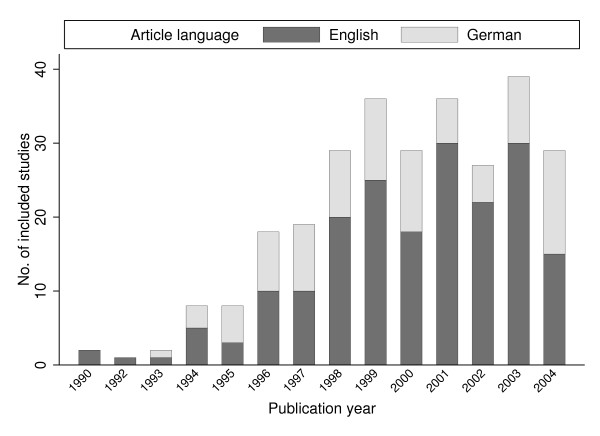
Included studies by article language and publication year.

As can be seen from figure 2, publication of German health economic studies has steadily increased until 1998 and has since then remained relatively stable with approximately 30 studies published per year. Of the included studies, 91 (32%) were published in German-language and no stable trend towards internationalization (in terms of publication in English-language) could be observed. The studies concentrate on few journals, and 134 out of 151 journals (89%) published less than three German economic evaluations between 1990 and 2004 (table 1). The vast majority of studies (79%) was published in medical journals in contrast to specialized health economic or health care sciences journals. The 91 German-language articles were published in 39 different German journals (37 medical and 2 health economics/services journals) with a median of 1 evaluation published per journal. Only 21 of these journals are listed in the ISI Journal Citation Reports.

Table 2 reports the characteristics of the included studies. Of the 283 studies that fulfilled all entry criteria, 0.4% were cost-benefit analyses, 6% cost-utility analyses, 14% cost-minimization analyses, 18% cost-effectiveness analyses with "life years gained" as outcome measure and 45% were cost-effectiveness analyses that used a clinical outcome as measure of effectiveness. Many studies (17%) were observational studies with information on costs that left health outcomes disaggregated and used no summary measure of benefit (cost consequence analyses). 20% of studies were multinational studies that evaluated health care in more than one country (Germany), usually in the context of other European nations.

**Table 1:** Distribution of included articles by journal and journal specialty

	1990–1998 (n = 87)		1999–2004 (n = 196)		1990–2004 (n = 283)	
	No. (%) studies		No. (%) studies		No. (%) studies	

By journal						
Pharmacoeconomics (E H)	10	(11.5)	18	(9.2)	28	(9.9)
Medizinische Klinik (G M)	9	(10.3)	6	(3.1)	15	(5.3)
Gesundheitsökonomie & Qual.mgmt (G H)	1	(1.1)	13	(6.6)	14	(4.9)
Deutsche Medizinische Wochenschrift (G M)	1	(1.1)	7	(3.6)	8	(2.9)
European Heart Journal (E M)	2	(2.3)	5	(2.6)	7	(2.5)
Other (n = 146 journals)*	64	(73.6)	147	(75.0)	211	(74.6)
By journal specialty						
Medical journals	70	(80.5)	154	(78.6)	224	(79.2)
Health economics/services journals	17	(19.5)	42	(21.4)	59	(20.9)

* Summarized over extracted categories; (E) English-language journal; (G) German-language; (M) Medical journal; (H) Health economics/services journal

**Table 2:** Characteristics of included studies

	1990–1998 (n = 87)		1999–2004 (n = 196)		1990–2004 (n = 283)	
	No. (%) studies		No. (%) studies		No. (%) studies	

By study type						
Cost minimization	16	(18.4)	23	(11.7)	39	(13.8)
Cost consequence	13	(14.9)	34	(17.4)	47	(16.6)
Cost effectiveness (life years gained)	17	(19.5)	35	(17.9)	52	(18.4)
Cost effectiveness (clinical outcome)	38	(43.7)	88	(44.9)	126	(44.5)
Cost utility	3	(3.5)	15	(7.7)	18	(6.4)
Cost benefit	0	(0)	1	(0.5)	1	(0.4)
By study design						
Alongside RCT	3	(3.5)	31	(15.8)	34	(12.0)
Modeling study	53	(60.9)	101	(51.5)	154	(54.4)
Combination RCT/Modeling	3	(3.5)	15	(7.7)	18	(6.4)
Observational	28	(32.2)	49	(25.0)	77	(27.2)
By study perspective (as stated by authors)						
Not stated	51	(58.6)	75	(38.3)	126	(44.5)
Third party payer (e.g., insurance)	32	(36.8)	83	(42.4)	115	(40.6)
Societal	4	(4.6)	24	(12.2)	28	(9.9)
Other (mainly 'patients')*	0	(0)	14	(7.1)	14	(5.0)
By disease category (ICD-9 category heading)						
Circulatory system	22	(25.3)	35	(17.9)	57	(20.1)
Neoplasms	13	(14.9)	23	(11.7)	36	(12.7)
Infectious and parasitic	12	(13.8)	20	(10.2)	32	(11.3)
Nervous system and sense organs	4	(4.6)	20	(10.2)	24	(8.5)
Endocrine, nutritional, metabolic	7	(8.1)	15	(7.7)	22	(7.8)
Other*	29	(33.3)	83	(42.3)	112	(39.6)
By level of care						
Curative	63	(72.4)	148	(75.5)	211	(74.6)
Rehabilitative	2	(2.3)	6	(3.1)	8	(2.8)
Preventive	22	(25.3)	42	(21.4)	64	(22.6)
By intervention type						
Pharmaceutical	49	(56.3)	96	(49.0)	145	(51.2)
Medical procedure	6	(6.9)	38	(19.4)	44	(15.6)
Screening	12	(13.8)	16	(8.2)	28	(9.9)
Other*	20	(23.0)	46	(23.5)	66	(23.3)

* Summarized over extracted categories

### Funding sources and disclosure of funding

**Figure 3:**
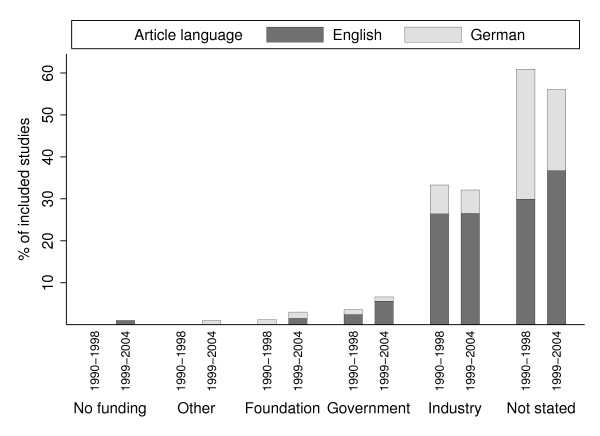
Included studies by funding statement, article language and publication year range.

Figure 3 shows the distribution of funding statements and different types of sponsorship organizations by year range. Among those studies that included a clear funding statement (n = 120) the fraction of industry-sponsored studies is very high (77%) and decreased only slightly (85% in 1990–1998 vs. 73% in studies published 1999–2004, p = 0.572). For studies that evaluated pharmaceutical products, this figure is even higher (91%). Declaration of sponsorship was strongly associated with journal specialty (35% medical journal articles disclosing funding vs. 69% articles in health economic journals, p < 0.001) and with article language (49% of English articles vs. 29% of German-language articles, p < 0.001). The fraction of German-language studies that declared funding increased from 23% in articles published between 1990 and 1998 to 32% of studies published between 1999 and 2004 (p = 0.340).

### Trends in published evaluations

As can been seen from the comparison of studies published prior to 1999 and those published between 1999 and 2004, there seems to be a systematic trend towards more comprehensive methods of evaluation and study designs. Articles published after 1998 were more likely to be conducted alongside randomized controlled trials at the expense of modeling and observational studies (4% vs. 16%, p = 0.009). We also observe differences in distribution of studies by primary ICD-9 category between the time periods. While the same five categories remain the most used ones, a distinct increase in studies evaluating care relating to nervous system and sense organ diseases results in a switch of ranks with the endocrine, nutritional and metabolic disease category. In addition, the later time period (1999–2004) is characterized by more diversity in studied ICD-9 categories as an increase in the "other" category documents. This variation may also be related to the strong increase in the assessment of medical procedures.

### Study perspective

In less than half of all studies the authors explicitly reported the perspective the study was undertaken from, but this fraction significantly increased over time (41% in 1990–1998 vs. 62% in 1999–2004, p < 0.001). Study perspective reporting practice was strongly associated with journal specialty (35% in medical journals vs. 69% in health care sciences journals, p < 0.001) and the observed shift is mainly attributable to changes in reporting practice in studies published in medical journals during the last years. However, not only the reporting of study perspective has significantly increased but also the distribution of the different perspectives chosen by investigators. Considering only those studies that included a clear statement on the chosen perspective (n = 157), the fraction of studies undertaken from the more comprehensive societal perspective rose from 11% in the first to 20% in the second time period (p = 0.032). Of the 28 studies claimed to be undertaken from the societal perspective, 18 were cost-effectiveness studies with clinical measures of effectiveness, 5 were cost-effectiveness studies with life years gained as outcome, 4 were cost-utility studies and the remaining was a cost-minimization study. This distribution indicates that the societal perspective manifested itself more frequently in a comprehensive costing methodology rather than in the societal valuation of benefits.

### Analysis of cost-utility studies

Among the 18 identified cost-utility-studies, 16 were published in medical journals of which 3 articles were in German-language and 2 were published in health economic journals. The methodology used to obtain utilities was very diverse and generally poorly reported. The vast majority of studies (n = 13) relied at least to some extent on secondary data but failed to provide even basic information on the methodology used in the secondary sources. In particular, most studies did not specify the original populations involved (e.g., sample size, country of origin, experts, patients or general public) and the relevant information was extracted by consulting the cited original sources. Four studies obtained primary data on patients' self-assessed quality-of-life or experts' assessment on behalf of their patients from small samples (*n *ranges between 22 patients and 70 experts). Quality-of-life data were then transformed into utilities using either disease category mapping to the Rosser-Matrix or empirically estimated transformations such as the Brazier function based on other nations' general population values [14]. Two modeling studies used a published transformation of disease-specific disability measures to time-trade off values originally obtained in a Canadian sample [15]. Two analyses referred to original studies that had used patients' quality-of-life assessment or experts' visual-analogue scale values from Sweden and Netherlands respectively and had applied a transformation or weighting ("social tariff") function based on health-related preferences from yet another country. Two studies used US-American values from multiple sources that were based on empirical analysis or expert judgment. Four studies relied completely on "judgment" without reference to secondary data or on published utility values for which it was unclear even from the original article whether these were empirically derived or based on judgment.

Four studies were classified as calculating quality-adjusted life years based on "primary data" of which three were modeling studies that referred to their quality-of-life data collection published elsewhere. Two of these articles published by the same working group report in their primary studies extensive quality-of-life assessment using different techniques in samples of 348 and 428 German patients. Utilities used for the base case of the analyses were derived by using patients' rating scale values and transforming these into utilities with the "Torrance" function which is based on preferences elicited via the standard-gamble in a sample of Canadian citizens [16]. Values obtained by other elicitation techniques and other samples (e.g., experts) had been used for sensitivity analyses. One study that referred to authors' primary data collection had elicited utilities in a sample of 21 German patients using the EuroQol instrument. However, only relative reductions in quality of life expressed as percentage points were documented. Finally, one analysis reported generation of primary data using the EQ-5D instrument (VAS and index) in samples of German patients. However, it is unclear how the authors estimated index values and which value set had been applied to calculate quality-adjusted life years. Measurements were obtained in two treatment arms and at multiple points in time and the respective sample sizes could not be determined.

In summary, in only three studies was the original methodology to obtain utilities well documented and comprehensible without consulting the original data sources. With the exception of one study, for which it could not be determined, no single study relied completely on German patients' or community health state preferences. Most concerning, a majority of studies mixed utilities elicited from various perspectives, nations and weighting approaches and did not actively disclose these sources of origin, e.g., by naming the populations in which preferences had originally been elicited.

## Discussion

To the authors' knowledge, this is the first study that provides a systematic overview of German health economic evaluative studies. We found that about a third of included evaluations were published in German-language and can thus be expected to be missing in international reviews. In a review of Spanish economic evaluations of health care covering also earlier years of publication, the majority of studies (77%) were published in local journals [17]. As Garcia-Altes we found that those national analyses that are published in local media are spread over a high number of journals and can predominantly be found in journals with a general bio-medical background. However, our key findings are in concordance with the results of international analyses of the literature. As others, we found that the majority of economic appraisals concentrate on few disease categories [18]. While these categories do reflect the current burden of disease in western societies, some important health areas have gained only limited attention [19]. For example, musculoskeletal, mental and pulmonary diseases are strongly underrepresented in the German economic evaluation literature though they bear a heavy burden that is even likely to increase in the future. Similarly, the fraction of studies that examined rehabilitative care is small and has remained stable over the past 14 years. These results may not only be explained by authors' research interests or awareness regarding certain areas of health and health care, but may also be related to the interests of funding bodies.

The fact that three quarters of all studies and nearly all studies evaluating pharmaceuticals that included a funding statement were industry-sponsored is concerning. The biases associated with industry-sponsorship have been acknowledged as being problematic internationally [20-24]. Bell et al. recently systematically assessed publication bias in health economic evaluations and found that most published analyses report favourable incremental cost effectiveness ratios below 20,000 $/QALY [25]. Studies funded by industry were more likely to report ratios below the three analyzed thresholds ((20,000 $/QALY adjusted odds ratio 2.1, 95% confidence interval 1.3–3.3), 50,000 $/QALY (3.2, 1.8–5.7), and 100,000 $/QALY (3.3, 1.6–6.8)). Compared to the international literature, the potential influence of industry-sponsoring in Germany seems more advanced. In an analysis of evaluations included in the HEED database that were published between 1992–2001, Pritchard reports that government and publicly funded policy making bodies are the most important source of funds being involved in 40% of studies, followed by the pharmaceutical industry at around one third [26]. As in our study, industry funding is more frequent in the evaluation of pharmaceuticals but at a considerable lower level (57% in studies published 1997–2001). This important role of the pharmaceutical industry in funding may in part explain why certain health problems or aspects of care delivery, e.g., rehabilitative care, are underrepresented in health economic studies. The finding that funding and publication bias is not only inherent in *what *is being evaluated but also associated with the *results *in terms of favorability of health care interventions calls the realistic value of health economic evaluations for decision makers seriously into question.

Our study indicates a systematic trend towards more comprehensive methods of evaluation, study perspectives and study designs. Though this result is encouraging, it has to be interpreted cautiously. "Comprehensiveness" is not in itself an exclusive goal and must be accompanied by methodological rigor in study design, model building, data collection and other aspects. However, as a limitation of our study, we extracted data on study characteristics and did not explicitly assess the quality of studies, e.g., by approving methods to calculate costs. For example, the observed increase in clear statements regarding study perspective can clearly be regarded a development towards *quality of reporting*. However, we did not qualitatively verify whether authors' statements regarding study perspective were justified. And even in case the labeling of perspective was justified, this does not necessarily imply that the chosen perspective itself was appropriate to the study question.

Regarding key aspects of methodology reporting in health economic studies, our study also confirms the findings of Neumann et al. who analyzed adherence to recommended reporting practice in English-language cost-utility studies published between 1976–1997 and 1998–2001 [1]. As Neumann et al., we observed an increase in authors' presentation of study perspective over the years. The fraction of studies that clearly report study perspective in German analyses published 1999–2004 (62%) is still considerably lower than in English-language international cost-utility analyses published 1998–2001 (73%), but much higher than the figure that has been reported for Spanish economic evaluations published 1969–1999 (27%) [17]. Also in concordance with Neumann et al., the frequency of statements of funding source disclosure in analyses pertaining to Germany remained stable over the years.

As reported for the international literature, there is extensive variation in the methodology used for health measurement and preference elicitation in German cost-utility studies [1,27-29]. Cost-utility-analysis is a methodological challenge and the techniques for utility elicitation and calculation of QALYs can be demanding and complex. Appropriate study design and reporting of methodology is therefore crucial, but has been questioned in reviews of the international literature [30]. Two key aspects can be concluded from our analysis: First, due to the diversity in methodology, quality-adjusted life years calculated in different studies are not comparable and compatible with each other. Second, the vast majority of studies cannot be termed as assessing health benefits from German patients' or the German population perspective. This is problematic since intercultural differences in health state preferences have been observed [31-34]. For example, Greiner et al. recently investigated differences in time-trade off (TTO) values for EuroQol health states between the German general population and the British "social tariff" published by Dolan et al. [35,36]. The authors report that German and British TTO values were significantly different for 30 out of 35 health states with the German values being systematically higher than the UK values. Most concerning, instead of basing the calculation of QALYs consistently on utilities that were obtained in a single European neighbor population as response to a lack of primary German data, many analyses included in our review mixed multiple sources. It can only be hoped for that recent efforts to estimate European and German "tariffs" will increase consistency in QALY calculations in the future [37,38]. Recent analyses of the international cost-utility literature suggest a trend towards the increased use of generic health status instruments and the elicitation of preferences in community samples [39]. Besides these considerations relating to study design itself, the adherence to recommended reporting practices remains a serious problem. As Richardson and Manca, who undertook a review of cost-utility-studies conducted alongside randomized clinical trials, we observed an alarming flaw in the reporting of key methodological aspects in cost-utility-studies [10]. Gerald et al. report in a review of international cost-utility analyses published in 1996 that in nearly 90% of studies, authors had clearly described how quality-of-life weights were assigned [30]. In contrast to this quite satisfactory figure, we often had to consult two and more secondary studies to gain insight into the very basic question of preference elicitation, namely: *Who has been asked what? *to derive utility weights. Due to a lack of transparency, e.g., through "chain referencing", many of the outlined deficits cannot easily be discovered by readers, in particular those unfamiliar with health economics. Given that the majority of studies are published in medical journals, and a further diffusion into medical subspecialty journals can be expected, responsibility of editors and peer-reviewers in the process of publishing is therefore essential [30,40,41]. Specialized health economic databases, such as the NHS-EED, OHE-HEED or the recently established European Network of Health Economic Evaluation Databases (EURONHEED) that include additional, standardized review of study methodology and data sources may become increasingly important tools to assess study quality after publication [42,43].

## Conclusion

Many of the German-language evaluations included in our study are likely to be missing in international reviews and may be systematically different from English-language reviews from Germany. Lack of transparency and adherence to recommended reporting practices constitute a serious problem in German economic evaluations.

## Competing interests

The author(s) declare that they have no competing interests.

## Authors' contributions

DLBS and TAB conceived of the study and participated in its design. Both authors conducted the literature review. TAB coordinated data retrieval and extraction. DLBS performed the statistical analysis and drafted the manuscript. Both authors read and approved the final manuscript.

## Pre-publication history

The pre-publication history for this paper can be accessed here:



## Supplementary Material

Additional File 1 Search strategies used. The table provides the details of search strings, combinations and delimiters of the literature search for each database approached. Click here for file
