# Supplementary material for: HEE-GER: a systematic review of German economic evaluations of health care published 1990–2004
Source: BMC Health Serv Res. 2007 Jan 12;7:7. doi: 10.1186/1472-6963-7-7 (PMC1781069; doi:10.1186/1472-6963-7-7)
Supplement: Additional File 1 — Search strategies used. The table provides the details of search strings, combinations and delimiters of the literature search for each database approached. [file 1472-6963-7-7-S1.pdf]

Additional file 1: Search strategies used

EMBASE (via interface of the "German Institute of Medical Documentation and Information", DIMDI)

#1 EM74

#2 (((((CT D "quality adjusted life year" OR UT="quality adjusted life year" OR IT="quality adjusted life year" OR SH="quality adjusted life year") OR (CT D "cost analysis" OR UT="cost analysis" OR IT="cost analysis" OR SH="cost analysis")) OR (CT D ("cost effectiveness analysis"; "cost consequence analysis"; "cost efficiency analysis"; "cost utility analysis"; "cost minimization analysis"; "cost benefit analysis") OR UT=("cost effectiveness analysis"; "cost consequence analysis"; "cost efficiency analysis"; "cost utility analysis"; "cost minimization analysis"; "cost benefit analysis") OR IT=("cost effectiveness analysis"; "cost consequence analysis"; "cost efficiency analysis"; "cost utility analysis"; "cost minimization analysis"; "cost benefit analysis") OR SH=("cost effectiveness analysis"; "cost consequence analysis"; "cost efficiency analysis"; "cost utility analysis"; "cost minimization analysis"; "cost benefit analysis"))) OR (CT D "economic evaluation" OR UT="economic evaluation" OR IT="economic evaluation" OR SH="economic evaluation")) OR (CT D ("cost effectiveness league table"; "cost effectiveness ratio"; "cost effectiveness") OR UT=("cost effectiveness league table"; "cost effectiveness ratio"; "cost effectiveness") OR IT=("cost effectiveness league table"; "cost effectiveness ratio"; "cost effectiveness") OR SH=("cost effectiveness league table"; "cost effectiveness ratio"; "cost effectiveness"))) OR (CT D "economic evaluation" OR UT="economic evaluation" OR IT="economic evaluation" OR SH="economic evaluation")

#3 (((FT=("Kosten Minimierungs Analys?"; "Kosten Minimierung?"; "Kosten Effektivität?"; "Kosteneffektivität?"; "Kosteneffizienz?"; "Kosteneffizienz?"; "Kosten Nutzwert?"; "Nutzwert"; "Kosten Nutzen?"; "Kosten Nutzen Analys?"; "Kostenanalys?"; "Kosten Analys?"; "Kosten") OR FT=("?konomisch? Evaluation?"; "?konomisch? Analyse?")) OR FT=("Gesundheitsökonomi? Evaluation"; "Gesundheitsökonomi? Analyse"; "Gesundheitsökonomi?"; "Pharmakoökonomi?"; "Qualitäts adjustiert?"; "Qualitätsadjustiert?"; "Cost minimi?")) OR FT=("Cost effective?"; "Cost efficiency?"; "cost efficiency analys?"; "cost effectiveness"; "cost analys?"; "cost effectiveness analysis"; "cost analy?"; "cost benefit analys?"; "cost utility analys?"; "economic? Evaluation")) OR FT=("Economic analys?"; "QALY"; "Quality adjusted life year?"; "Economic assessment"; "Health Economic?"; "Pharmacoeconomic?")

#4 2 OR 3

#5 (CT D ("german federal republic"; "german democratic republic"; "germany") OR UT=("german federal republic"; "german democratic republic"; "germany") OR IT=("german federal republic"; "german democratic republic"; "germany") OR SH=("german federal republic"; "german democratic republic"; "germany")) OR FT=("Deutschland"; "Germany"; "deutsch?"; "German?")

#6 (CT D "article" OR UT="article" OR IT="article" OR SH="article")

#7 4 AND 5 AND 6

#8 PPS=human

#9 7 AND 8

#10 (9) AND PY=1990 to 2004 AND (LA=german OR LA=english)

PUBMED

("1990"[PDAT] : "2004"[PDAT])

NOT (Letter[ptyp] OR Editorial[ptyp] OR review[ptyp])

AND ("germany"[MeSH Terms] OR German[Text Word])

AND (cost effectiveness[text word] OR cost effective[text word] OR cost utility[text word] OR economic evaluation[text word] OR Cost consequence[text word] OR Cost minimization[text word] OR Cost minimisation[text word] OR Cost efficiency[text word] OR Cost benefit[text word] OR Quality adjusted life year[text word] OR Economic analysis[text word] OR Cost analysis [text word])

ECONLIT (silverplatter)

#1 Health in DE

#2 Freetext (AB, AF, AU, AV, BK, BR, DE, FS, GE, NA, PB, RF, SO, TI) German*

#3 PY=1990-2004

#4 DT=journal-article

#5 #1 AND #2 AND #3 AND #4 AND #5

CINAHL direct

#1 TW[textword search]: German*
#2 Explode: Cost-benefit-analysis.mjx OR Costs.mjx
#3 #1 AND #2
AND Limit publication type: journal-article.pt.
AND Limit year: 1990-2004
AND Limit language: English OR German

NHS-Pharmline

#1 Freetext: (German* AND ("Costs" OR "QALY" OR "quality-adjusted life year" OR "Cost Analysis" OR "Cost Utility" OR "Cost Minimisation" OR "Cost Minimization" OR "Cost Effectiveness" OR "Cost Benefit" OR "Cost consequence" OR "Pharmacoeconomic" OR "Economic evaluation" OR "Economic analysis))
AND Year delimiter: 1990 to 2004

NHS EED

All fields: German
AND Date of publication: 1990 OR 1991 OR 1992 OR 1993 OR 1994 OR 1995 OR 1996 OR 1997 OR 1998 OR 1999 OR 2000 OR 2001 OR 2002 OR 2003 OR 2004
AND Record type: Economic evaluations

OHE HEED (Expert Search)

AX[All data]: German OR Germany
AND JD[Journal date] 1990 OR 1991 OR 1992 OR 1993 OR 1994 OR 1995 OR 1996 OR 1997 OR 1998 OR 1999 OR 2000 OR 2001 OR 2002 OR 2003 OR 2004
